# From Weed Evolution to Crop Design: A Computational Blueprint for a Novel, Synergistic Herbicide-Resistant Allele in Wheat

**DOI:** 10.3390/plants15132023

**Published:** 2026-06-30

**Authors:** Yuexing Wang, Qinge Chen, Zhangpeng Shi, Tian Mi, Yujiu Wu, Na Niu, Lingjian Ma

**Affiliations:** College of Agronomy, Northwest A&F University, Yangling 712100, China1391489154@nwafu.edu.cn (Q.C.); shizhangpeng01@163.com (Z.S.); 18734253981@163.com (T.M.);

**Keywords:** herbicide resistance, predictive crop design, acetolactate synthase, mesosulfuron-methyl, molecular dynamics, wheat breeding

## Abstract

The escalating crisis of herbicide-resistant weeds threatens global wheat production. While key mutations are well-documented in weeds, the principles governing their interactions in wheat remain largely unknown. Here, we first developed a novel wheat germplasm carrying the acetolactate synthase (*TaALS*) Ser-627-Asn (S627N) mutation via ethyl methanesulfonate (EMS) mutagenesis. We then employed a computational design strategy to explore its synergy with the prevalent Trp-548-Leu (W548L) mutation—a combination not yet reported in nature. Integrated molecular dynamics (MD) simulations and free energy landscape analysis revealed that the in silico W548L/S627N double mutant triggers synergistic global destabilization of the herbicide–enzyme complex. Binding affinity progressively weakened from wild-type (−25.54 ± 2.05 kcal/mol) to the double mutant (−18.13 ± 2.76 kcal/mol), driven by a polarity inversion at the Arg-347 anchor. Comparative transcriptomic profiling of the S627N germplasm confirmed the absence of deleterious metabolic feedback in the branched-chain amino acid biosynthesis pathway. This work exemplifies a paradigm shift from mimicking natural variation to predictive crop design via multiplex gene editing.

## 1. Introduction

Weed competition continues to be one of the toughest biotic stresses facing global wheat production. In heavily infested fields—especially those under intensive cereal monoculture—yield losses commonly range from 20% to over 50% [1,2,3]. Grass weeds that are phylogenetically close to wheat, such as *Aegilops tauschii* (the D-genome donor) and *Alopecurus aequalis*, create particular problems. They occupy very similar ecological niches, look and grow alike, and share overlapping phenology, which makes selective chemical control extremely difficult [4,5,6,7,8,9].

Acetolactate synthase (ALS)-inhibiting herbicides, particularly sulfonylureas like mesosulfuron-methyl (MSM), have long been a cornerstone for managing these weeds in winter wheat systems across China and many other major wheat-growing regions [10,11,12,13]. But their long-term usefulness is now under serious threat from two opposing trends. On one side, quite a few elite wheat cultivars show variable—and sometimes quite severe—phytotoxicity, depending on genotype sensitivity, environmental conditions, and application timing [10,12,13]. On the other side, resistance in target weed populations has escalated rapidly worldwide, driven by both target-site and non-target-site mechanisms [14,15,16,17,18,19,20,21,22,23,24,25].

Ironically, this growing resistance crisis has also given us a valuable library of naturally evolved *ALS* alleles. Target-site substitutions at conserved positions, such as Pro-197 and Trp-574, are dominant resistance mechanisms [26,27,28]. In hexaploid wheat (*Triticum aestivum*), homeologous gene dosage complicates the direct importation of weed-derived alleles. A forward-looking approach is to extract biophysical rules from weed resistance to redesign alleles tailored to wheat’s genomic background [20,28].

Even more striking is the increasing evidence of synergistic interactions—either between multiple target-site changes or combined with enhanced metabolic detoxification—that can push resistance levels well over 100-fold in field-evolved populations [17,18,23,29,30]. These allele combinations reveal a very refined evolutionary trade-off: strong resistance with relatively low fitness costs—something conventional crop breeding has struggled to replicate consistently in polyploid genomes [31].

In hexaploid wheat, simply importing these weed-derived alleles is usually not optimal due to subgenome-specific expression patterns and epistatic interactions absent in diploid weeds [32]. A better approach is to extract the biophysical rules behind weed resistance and then rationally redesign alleles tailored to wheat’s complex genomic background [33]. By crop design, we refer to the rational engineering of crop genomes to achieve desired agronomic traits, as opposed to traditional breeding or random mutagenesis. Predictive crop design extends this concept by using computational models (e.g., molecular dynamics simulations, free energy calculations) to forecast the phenotypic outcomes of specific genetic modifications before experimental validation. The functional wheat equivalents of the major weed resistance hotspots are Trp-548-Leu (W548L) and Ser-627-Asn (S627N) [32]. W548L has already been well documented as a strong resistance allele in wheat mutant screens [31,32], but its potential synergistic interaction with S627N—and the atomistic basis of any such synergy—has never been explored.

To explore this opportunity, we developed an integrated “design–test–learn” pipeline. We first utilized an ethyl methanesulfonate (EMS)-mutagenized wheat germplasm carrying the *TaALS-6D* S627N mutation. The resistance phenotype and physiological characteristics of this line (e.g., *GR*_50_ values and biochemical responses under MSM stress) were previously characterized and validated through whole-plant dose–response assays in our laboratory [34]. Building on this empirical foundation, we applied AlphaFold3 and all-atom molecular dynamics (MD) simulations (in silico, 100 ns replicates) to rationally design and dissect the previously unreported W548L/S627N double mutant. Our results reveal an “energy network reprogramming” mechanism that destabilizes the herbicide–enzyme complex while preserving catalytic competence. This work therefore provides not only an immediately usable genetic resource (the S627N germplasm) but also a predictive, atomistic blueprint for engineering durable, epistasis-informed herbicide tolerance in polyploid crops [35,36,37].

## 2. Results

### 2.1. Global Herbicide Resistance Trends and ALS Mutation Hotspots

**Figure 1:**
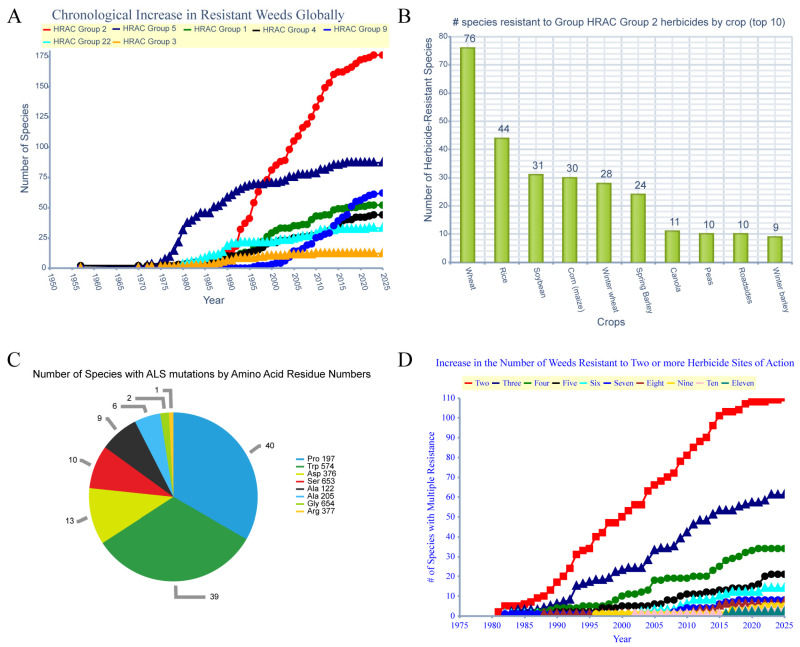
Global landscape of herbicide resistance evolution and the dominance of *ALS* target-site mutations. (**A**) Temporal accumulation of unique resistant weed biotypes classified by herbicide mode of action (HRAC Group). Resistance to ALS inhibitors (Group 2) shows the steepest trajectory, representing the most prevalent resistance category globally. (**B**) Distribution of weed species resistant to HRAC Group 2 herbicides across major crops. Wheat systems harbor the highest diversity of resistant species, reflecting intense selection pressure from reliance on sulfonylurea herbicides. (**C**) Frequency distribution of resistance-conferring amino acid substitutions across weed species. Substitutions at Pro-197 and Trp-574 emerge as the predominant evolutionary solutions for survival under herbicide stress. (**D**) Chronological increase in the number of weed species evolving multiple herbicide resistance (MHR) to two or more sites of action, highlighting the growing threat of multi-resistant populations.

Herbicide resistance has escalated into a global crisis for agricultural productivity. As of 2025, the International Herbicide-Resistant Weed Database documents more than 539 unique resistant biotypes across 102 crops in 75 countries. Notably, resistance to ALS-inhibiting herbicides (HRAC Group 2) exhibits the steepest accumulation trajectory, making it the most prevalent resistance category worldwide (Figure 1A). Wheat production systems represent a primary epicenter of this crisis, harboring the highest diversity of ALS-inhibitor-resistant weed species (Figure 1B). This disproportionate burden underscores the extreme vulnerability of wheat cultivation to the failure of single-mode-of-action herbicides. At the molecular level, target-site mutations within the *ALS* gene constitute the predominant resistance mechanism. Global surveillance data highlight specific “evolutionary hotspots”: substitutions at Pro-197 and Trp-574 are the most frequent, documented in 40 and 39 weed species, respectively (Figure 1C). The Trp-574-Leu substitution, in particular, is a definitive mechanism conferring high-level resistance to sulfonylureas such as mesosulfuron-methyl. Compounding this challenge, the accelerating occurrence of weeds resistant to multiple herbicide sites of action signals a shift toward increasingly complex resistance genotypes (Figure 1D). Nevertheless, these naturally evolved mutations also offer a solution. Refined by intense selection pressure, they pinpoint critical residues that tolerate herbicide binding while preserving enzymatic function. This evolutionary insight supports a reverse-genetics strategy: translating these validated “design templates” from weeds into crop genomes. Our study specifically targets the high-value Trp-548 and Ser-627 sites in wheat *TaALS*—the functional homologs of the widespread Trp-574 and Ser-653 mutations in weeds—to engineer robust herbicide tolerance.

### 2.2. Identification of the Causative S627N Mutation via Multi-Omics Sequencing

**Figure 2:**
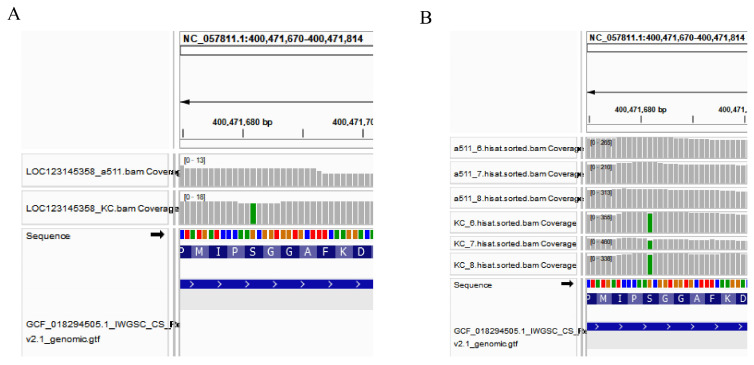
Multi-omics validation identifies the causal SNP in *TaALS-6D*. (**A**) Genomic identification. IGV visualization of whole-genome resequencing data reveals a specific SNP in LOC123145358 (*TaALS-6D*) in the resistant KC line compared with the sensitive a511 line. (**B**) Transcriptomic verification. RNA-seq alignment confirms that the mutation is actively expressed in the transcriptome, demonstrating stable transcription of the mutant allele.

To decipher the genetic basis of MSM resistance, we performed comparative whole-genome resequencing of the sensitive (a511) and resistant (KC) wheat lines. This analysis pinpointed a pivotal single-nucleotide polymorphism (SNP) within the *TaALS-6D* gene (LOC123145358). The mutation involves a G-to-A transition that results in a Ser-627-Asn substitution in the encoded protein (Figure 2A). Crucially, transcriptome sequencing and visualization via IGV corroborated that this genomic alteration is stably maintained and actively transcribed at the cDNA level, confirming expression of the mutant allele in the resistant germplasm (Figure 2B).

### 2.3. Phylogenetic Validation of TaALS-6D as the Functional Herbicide Target

**Figure 3:**
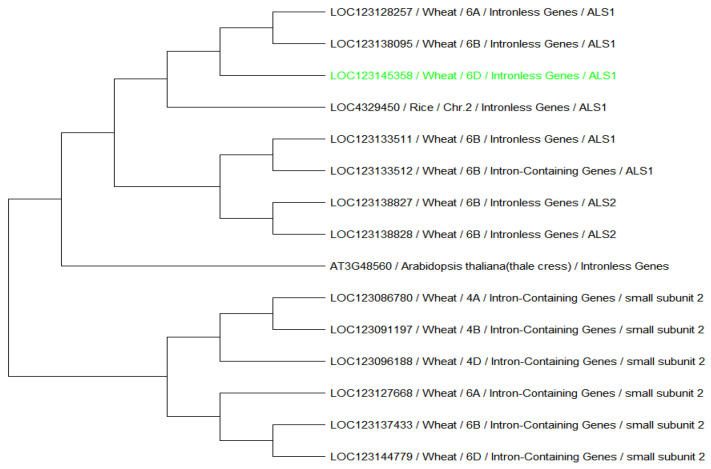
Phylogenetic reconstruction identifies *TaALS-6D* as a canonical intronless *ALS1* ortholog. The Neighbor-Joining tree classifies wheat *ALS* family members into two distinct clades: intronless genes (*ALS1*/*ALS2*) and intron-containing genes (small subunits). LOC123145358 (*TaALS-6D*, marked in green) clusters with its A- and B-subgenome homeologs and the rice *ALS1* ortholog (LOC4329450), confirming its identity as a functional catalytic subunit.

To distinguish the functional resistance locus from other members of the complex wheat ALS family and confirm its identity as the canonical herbicide target, we conducted a systematic phylogenetic analysis of all annotated ALS-related sequences. The phylogenetic reconstruction resolved the family into two architecturally distinct clades: the intron-containing small subunits and the intronless catalytic subunits (ALS1)—the latter being the bona fide target site of sulfonylurea herbicides (Figure 3). As shown in Figure 3, LOC123145358 clusters firmly within this intronless ALS1 clade, grouping with its homeologs and the rice ALS1 ortholog (LOC4329450). This phylogenetic placement confirms that LOC123145358 (hereafter TaALS-6D) encodes a functional catalytic subunit sensitive to herbicide inhibition, rather than a regulatory isoform. Furthermore, the S627N mutation maps to a highly conserved domain in the ALS protein that has been previously identified as a key determinant of sulfonylurea herbicide resistance. These findings definitively establish TaALS-6D as the critical, herbicide-targeted member of the wheat ALS family responsible for conferring resistance [32].

### 2.4. Atomistic Dissection of Synergistic Resistance Mechanisms

#### 2.4.1. The ‘Dual-Arginine Anchor’ Defines the High-Affinity Binding Mode of Wild-Type TaALS

**Figure 4:**
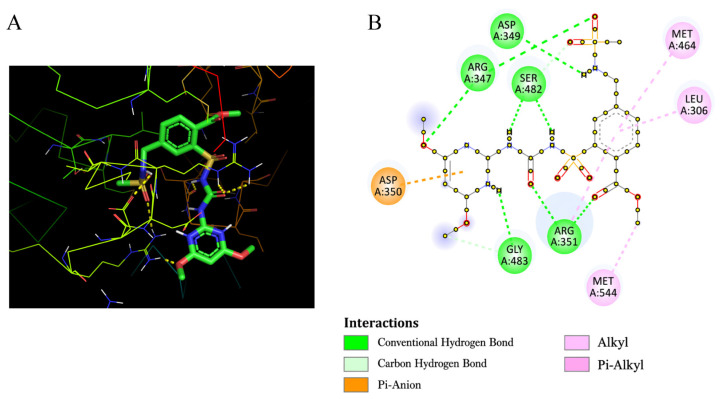
Molecular docking reveals the “Dual-Arginine Anchor” mechanism in wild-type TaALS. (**A**) Three-dimensional binding pose of mesosulfuron-methyl (MSM) in the catalytic pocket. (**B**) Two-dimensional interaction diagram highlighting the primary stabilizing forces: the pivotal hydrogen-bond network with the dual-arginine anchor (Arg-347, Arg-351) and auxiliary Pi-alkyl/hydrophobic interactions with Leu-306, Met-464, and Met-544.

Molecular docking simulations resolved the high-affinity binding architecture of MSM within the wild-type *TaALS* active site (Figure 4). The herbicide is stabilized by a dense intermolecular network, anchored primarily by the conserved “dual-arginine” motif. Specifically, the sulfonylurea bridge forms critical hydrogen bonds and electrostatic interactions with Arg-347 and Arg-351, effectively locking MSM at the entrance of the catalytic channel and sterically occluding substrate access. Beyond this polar anchor, the pyrimidine and phenyl rings engage in stabilizing Pi-alkyl and hydrophobic interactions with residues such as Leu-306, Met-464, and Met-544 (Figure 4B). This multivalent interaction network establishes the structural baseline for interrogating the destabilizing effects of resistance mutations.

#### 2.4.2. Mutations Compromise Structural Integrity and Complex Stability MD

**Figure 5:**
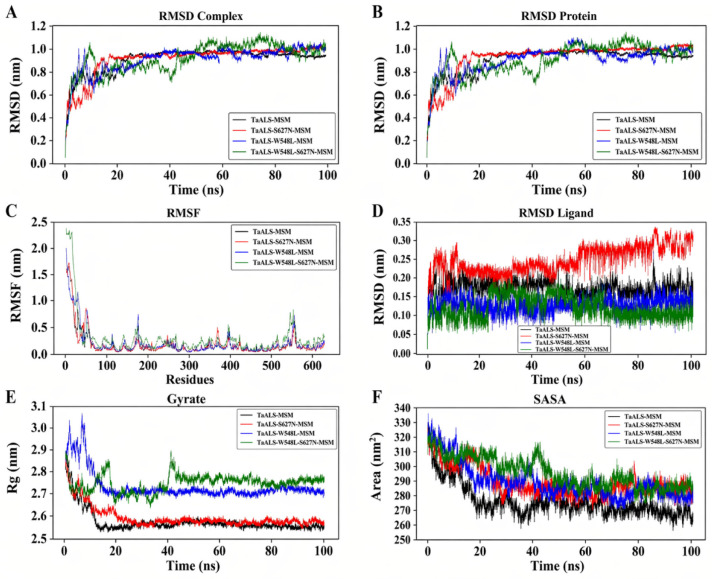
Synergistic destabilization of the TaALS–MSM complex in the double mutant. (**A**,**B**) Time-dependent RMSD of the complex (**A**) and protein backbone (**B**), showing significant conformational drift in the double mutant (green) compared with the stable wild-type (black). (**C**) RMSF profiles highlighting regions of induced backbone plasticity (flexibility) in the mutants. (**D**) Ligand RMSD revealing the positional instability of MSM within the mutant binding pockets. (**E**) Radius of gyration (Rg) indicating global structural expansion in the double mutant. (**F**) Solvent-accessible surface area (SASA) demonstrating increased solvent exposure and loss of hydrophobic-core integrity.

MD simulations (100 ns) were employed to interrogate the time-resolved structural evolution of the mutant complexes. The wild-type TaALS-MSM complex maintained a hyper-stable architecture, reaching a rapid equilibrium with minimal fluctuations in Root-Mean-Square Deviation (RMSD) for both the complex and protein backbone (Figure 5A,B). This indicates a rigid scaffold essential for high-affinity ligand locking.

In stark contrast, the mutant systems displayed continuous conformational drift. While the single mutants (S627N, W548L) showed moderate deviations, the W548L/S627N double mutant exhibited the most profound instability, failing to converge into a stable state. Root-Mean-Square Fluctuation (RMSF) analysis revealed that this global instability is driven by heightened plasticity in specific loop regions, which are significantly more flexible in the double mutant than in the wild-type (Figure 5C). Consequently, the MSM ligand itself lost its stable anchor, exhibiting erratic positional fluctuations within the double mutant’s binding pocket (Figure 5D).

This dynamic disorder culminates in a global structural expansion. The double mutant trajectory underwent a significant dilation, characterized by an increase in the Radius of Gyration (Rg) from ~2.60 nm (WT) to ~2.75 nm (Figure 5E). Crucially, this expansion compromised the hydrophobic core, as evidenced by the Solvent Accessible Surface Area (SASA) peaking at ~290 nm^2^ (Figure 5F). Collectively, these data demonstrate that the W548L and S627N mutations act synergistically to disrupt the enzyme’s compactness, allowing solvent intrusion and destabilizing the herbicide-binding interface.

#### 2.4.3. Energetic Destabilization and Network Remodeling Revealed by FEL MM/PBSA Calculations

**Figure 6:**
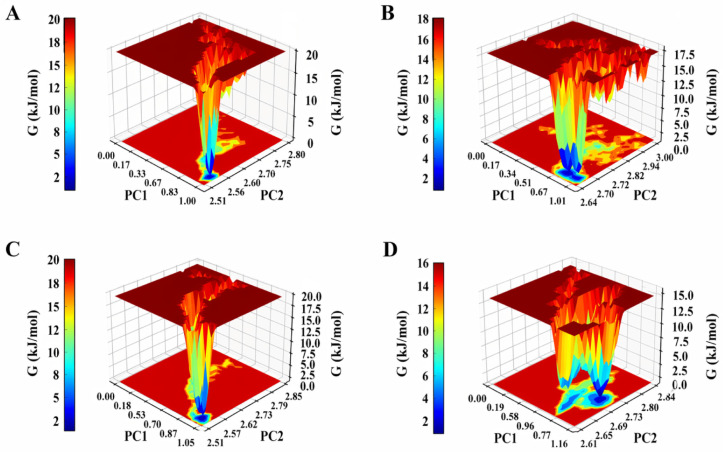
Conformational free energy landscapes (FEL) reveal thermodynamic destabilization. The FEL plots were generated by projecting MD trajectories onto the first two principal components (PC1 vs. PC2). The color bar represents the Gibbs free energy level (kJ/mol), with deep blue indicating stable low-energy basins. (**A**) Wild-type: Exhibits a single, concentrated deep basin, reflecting a rigid and stable lock-and-key conformation. (**B**) S627N and (**C**) W548L: Show broader basins indicating increased structural flexibility. (**D**) W548L/S627N Double Mutant: Displays a rugged landscape with a dispersed, shallow basin. This indicates a high-entropy state where the protein frequently samples open/unstable conformations incompatible with tight ligand binding.

MM/PBSA calculations showed progressive weakening of binding affinity from wild-type to the double mutant (Table 1). To visualize the thermodynamic consequences, we constructed Free Energy Landscapes (FEL). The wild-type complex occupied a single, deep energy basin, indicative of a stable, lock-and-key conformation (Figure 6A). In contrast, the in silico W548L/S627N double mutant sampled a broad, shallow basin with multiple metastable states (Figure 6D), reflecting a dramatic increase in conformational entropy that precludes stable herbicide binding.

#### 2.4.4. Per-Residue Analysis Uncovers an ‘Energy Network Reprogramming’ Mechanism

Decomposition of binding free energy to per-residue contributions revealed the atomistic driver of synergy (Supplementary Figure S1). While single mutants largely preserved favorable interactions at key residues such as Arg-347 and Arg-351, the double mutant exhibited a dramatic polarity inversion at Arg-347 and de novo energetic penalties at His-326 and Gln-463. This demonstrates that resistance arises not from a simple loss of contacts, but from a systemic reprogramming of the binding energy network.

### 2.5. The S627N Germplasm Maintains Metabolic Homeostasis Underlying Viable Resistance

Transcriptomic analysis was performed on field-grown plants treated with three times the recommended field dose of MSM to assess metabolic responses under strong herbicide selection pressure. To evaluate whether the S627N mutation incurs a fitness cost by disrupting core metabolism, we performed comparative transcriptomics of the resistant (KC) and susceptible (a511) lines. KEGG (Kyoto Encyclopedia of Genes and Genomes) pathway enrichment analysis highlighted ‘Amino acid metabolism’ among the altered categories. However, detailed inspection of the valine, leucine, and isoleucine biosynthesis pathway (Supplementary Figure S2) revealed a balanced transcriptional landscape. While specific genes exhibited compensatory fluctuations, the pathway maintained robust overall activity without evidence of systemic shutdown. Furthermore, comparative expression analysis showed no significant feedback induction of the TaALS gene family members in the mutant relative to the wild-type, as all tested genes exhibited comparable expression levels between genotypes (Supplementary Figure S3). This indicates that the S627N mutation preserves sufficient enzymatic function to avoid triggering deleterious compensatory responses, thereby explaining the germplasm’s normal growth.

## 3. Discussion

This study outlines a complete translational pipeline—from understanding naturally evolved weed resistance to designing predictive crop traits—that combines forward genetics, multi-omics validation, and high-resolution computational modeling to push forward herbicide-tolerant wheat breeding. By bridging EMS-derived germplasm creation with in silico rational design, we developed a novel S627N wheat line and dissected the mechanistic basis of the previously unreported synergistic W548L/S627N double mutant in TaALS. This provides both a practical breeding asset and new insight into epistatic resistance dynamics [29,33].

The S627N single mutant in the KC line stands out as particularly promising for wheat improvement. Whole-genome resequencing and RNA-seq pinpointed a causative G-to-A transition in TaALS-6D (LOC123145358), with transcriptomic data confirming stable expression of the mutant allele [13,29]. Phylogenetic analysis across the wheat ALS family clearly positioned TaALS-6D as the primary catalytic subunit targeted by sulfonylurea herbicides—consistent with earlier findings that D-subgenome mutations often deliver the strongest resistance phenotypes in wheat EMS populations [1,32]. Most importantly, comparative transcriptomics showed no systemic disruption in the branched-chain amino acid biosynthesis pathway and no compensatory upregulation of TaALS family members (Supplementary Figure S3), suggesting that enzymatic function is largely maintained without detectable fitness costs [29,31,32]. This kind of metabolic robustness is rare and highly desirable in herbicide-resistance breeding, where target-site mutations typically carry penalties in growth, yield, or competitive ability [33,37].

Interestingly, our transcriptomic comparison with the wild-type (a511) showed no obvious systemic disturbance in BCAA biosynthesis and no compensatory upregulation of TaALS genes in the S627N mutant. This points to preserved enzymatic function at the molecular level without detectable fitness costs, making the KC germplasm especially attractive for breeding programs. By contrast, target-site mutations in grass weeds frequently come with measurable fitness penalties—for example, ACCase substitutions in *Beckmannia syzigachne* have been shown to reduce growth rate and competitive ability under field conditions [38]. Similar trade-offs appear in other species as well: ACCase mutations in *Avena sterilis* populations significantly lowered seed production and biomass [39]; Ile-1781-Leu and Asp-2078-Gly mutations in *Lolium rigidum* resulted in shorter plants and delayed flowering [40]; and herbicide-resistant *Brachypodium hybridum* displayed environment-dependent fitness penalties, including reduced survival under drought stress [37]. These cases illustrate that weed mutations often involve trade-offs, whereas the hexaploid wheat genome’s built-in redundancy appears to buffer such negative effects—a pattern also supported by the minimal fitness impacts seen in ALS-edited wheat lines [31,32]. The metabolic stability we observe in the S627N germplasm therefore highlights its strong potential for developing sulfonylurea-tolerant varieties without sacrificing agronomic performance.

The S627N germplasm shows striking similarity at the conserved residue (equivalent to Ser-653-Asn in Arabidopsis) to naturally selected weed alleles, which have repeatedly served as effective templates for ALS-inhibitor tolerance in grass weed populations [1,4,18,20,21,30]. By adapting and optimizing these alleles within the hexaploid wheat genome—while carefully accounting for homeologous interactions and subgenome-specific effects—we can move beyond simple mimicry toward intentional, crop-tailored resistance design [33].

Building on these experimental foundations, our in silico examination of the previously unreported W548L/S627N combination uncovered a strong epistatic mechanism we term “energy network reprogramming.” MD trajectories, free energy landscapes, and per-residue decomposition consistently showed global conformational destabilization of the TaALS–MSM complex. This was primarily driven by polarity inversion at the critical Arg-347 anchor point, together with emergent energetic penalties at distal residues, resulting in a marked reduction in herbicide affinity (ΔG shift from −25.54 to −18.13 kcal/mol) while still preserving sufficient structural integrity for catalysis [25,26,27,33]. These findings go beyond the classical lock-and-key inhibition model and illustrate how non-additive network-level effects can produce high-level resistance with relatively low functional trade-offs [21,22,25,26].

We want to be clear about evidence strength: the S627N resistance phenotype is firmly supported by whole-plant resistance assays, multi-omics validation, and MSM exposure responses [13,29], whereas the W548L/S627N synergy remains a high-confidence computational prediction. That said, recent advances in wheat genome editing—such as efficient Cas12i3-mediated multiplex editing [35] and versatile prime editing [36]—now make experimental validation of double mutants realistic within a reasonable timeframe. Moreover, China’s evolving biosafety framework for gene-edited crops—including recent approvals for TaALS-modified wheat lines conferring ALS-inhibitor tolerance [41]—provides timely and supportive regulatory pathways for commercialization [33].

From a theoretical perspective, this work enriches current models of herbicide resistance evolution by highlighting epistatic interaction networks as major drivers of durable, high-level phenotypes under intense selection [21,22]. On the practical side, it points toward a shift in wheat breeding strategy: moving from reactive adaptation to proactive, simulation-guided, epistasis-aware trait engineering. The S627N germplasm already offers immediate sulfonylurea tolerance with low fitness cost [29,33], and the predicted W548L/S627N allele holds strong promise for greater durability via synergistic effects. By drawing inspiration from weed evolutionary innovation while overcoming its limitations, this integrated forward-genetics and computational approach lays a solid foundation for developing next-generation, resilient herbicide-tolerant varieties in polyploid cereals [33,35,36].

Similar rational ALS editing strategies have achieved low-cost herbicide resistance in diploid rice via the novel G628W allele, verifying that this integrated multi-omics + computational pipeline is broadly applicable across monocot staple crops beyond hexaploid wheat [42].

## 4. Materials and Methods

### 4.1. Field Experiment and Transcriptomic Analysis

Two wheat lines were used in this study: the susceptible wild-type line ‘a511’ (sensitive to mesosulfuron-methyl, MSM) and the resistant mutant line ‘KC’ (carrying the *TaALS-6D* S627N mutation). ‘a511’ is a conventional elite winter wheat cultivar widely grown in the Huang–Huai wheat region of China and served as the wild-type sensitive control. The ‘KC’ line was isolated from an EMS-mutagenized M_3_ population of ‘a511’ and was selected because it exhibited stable, high-level resistance to MSM while maintaining normal agronomic performance and no detectable fitness cost.

Whole-plant resistance assays were performed under controlled greenhouse conditions (22/18 °C day/night, 16 h photoperiod) by Lei Tianzhen (as detailed in [36]⁠). The S627N resistance phenotype was evaluated through dose–response assays following MSM application, including determination of GR_50_ values and visual injury assessment.

To evaluate the transcriptional response under realistic agronomic conditions, the two wheat lines (a511 and KC) were planted in the field at the experimental station of Northwest A&F University (Yangling, Shaanxi, China) following standard local practices. The seeds were sown in December 2024. When the seedlings reached the 3–4 leaf stage, they were sprayed with mesosulfuron-methyl (MSM) at three times the recommended field dose (40.5 g a.i. ha^−1^). Leaf tissues were collected from three biological replicates per genotype at 14 days after treatment, immediately frozen in liquid nitrogen, and stored at −80 °C until RNA extraction. Total RNA was extracted using a commercial plant RNA extraction kit (e.g., TRIzol reagent). RNA quality and integrity were verified by agarose gel electrophoresis and spectrophotometry (NanoDrop). Strand-specific cDNA libraries were constructed and sequenced on the Illumina NovaSeq 6000 platform (Novogene Co., Ltd., Beijing, China) using paired-end sequencing.

Genomic DNA was extracted from fresh leaf tissue using the CTAB method. To identify candidate mutations, whole-genome resequencing (WGS) and transcriptome sequencing (RNA-seq) were performed on the Illumina NovaSeq 6000 platform (Novogene Co., Ltd., Beijing, China). Sequencing generated approximately 30× genomic coverage and 50 million paired-end reads per transcriptome sample, ensuring high-confidence variant calling.

Raw sequencing reads (RNA-seq) were processed as follows: adapter sequences and low-quality bases were trimmed using Trimmomatic (v0.39). Clean reads were then aligned to the *Triticum aestivum* reference genome (IWGSC RefSeq v2.1) using HISAT2 (v2.2.1). Gene expression quantification was performed using StringTie (v2.2.1), and the resulting read counts were normalized to transcripts per million (TPM) values. Differential gene expression analysis between KC and a511 lines was performed using the DESeq2 package (v1.38.3) in R (v4.3.2), with genes having an absolute log_2_ fold change >1 and adjusted *p*-value (FDR) < 0.05 considered significantly differentially expressed. For functional interpretation, KEGG pathway enrichment analysis was conducted on the set of differentially expressed genes using the clusterProfiler package (v4.6.2). Enrichment significance was determined by hypergeometric test with FDR correction. Specific pathway visualization (e.g., valine, leucine, and isoleucine biosynthesis, map00290) and heatmap generation were performed using custom R scripts based on normalized expression values (Z-score transformed). The expression levels of all annotated TaALS gene family members were extracted from the overall expression matrix and compared between genotypes using Student’s *t*-test (*n* = 3 biological replicates).

### 4.2. Phylogenetic Analysis

Amino-acid sequences of 13 putative ALS family members were retrieved from the *Triticum aestivum* reference genome (IWGSC RefSeq v2.1) via Ensembl Plants. To determine orthology, reference ALS sequences from *Arabidopsis thaliana* (*AtALS*) and *Oryza sativa* (*OsALS*) were included. Multiple-sequence alignments were performed using ClustalW with default parameters. A phylogenetic tree was reconstructed using the Neighbor-Joining (NJ) method in MEGA 11. Evolutionary distances were computed using the Poisson correction method, and the reliability of internal branches was assessed with 1000 bootstrap replicates.

### 4.3. Protein Structure Prediction and Validation

The full-length sequence of TaALS-6D (LOC123145358) was used as the template. Three-dimensional structures for the wild-type and three mutant variants (W548L, S627N, W548L/S627N) were predicted using the AlphaFold3 engine. Only models exhibiting high per-residue confidence scores (pLDDT > 90) were selected for downstream analysis. The stereochemical quality of the predicted models was rigorously validated using Ramachandran-plot analysis to ensure that no residues fell into disallowed regions.

### 4.4. Molecular Docking

The chemical structure of MSM was retrieved from PubChem (CID 11409499; https://pubchem.ncbi.nlm.nih.gov/compound/11409499, accessed on 28 June 2025) and energy-minimized. The ligand was docked into the TaALS active site, with the binding-pocket coordinates defined based on the orthologous crystal structure of the Arabidopsis ALS–sulfonylurea complex (PDB ID: 1YBH). Semi-flexible docking was performed using AutoDock Vina 1.2.0. To ensure sampling convergence, the exhaustiveness was set to 64, generating 20 binding modes per run. The binding pose with the lowest affinity energy and highest consensus with known structure–activity relationship data was selected for simulation. Visualization was performed using PyMOL v3.1 and Discovery Studio Visualizer v2021.

### 4.5. MD Simulations and Free Energy Calculations

All-atom MD simulations were conducted using GROMACS 2022.3. The protein topology was generated with the AMBER99SB-ILDN force field, while the MSM ligand was parametrized using Sobtop v1.0-dev2(GAFF-compatible). Systems were solvated in a dodecahedral box with TIP3P water molecules and neutralized with counter-ions (Na^+^/Cl^−^). Following energy minimization (steepest descent), the systems underwent two-stage equilibration: NVT (canonical ensemble) and NPT (isothermal–isobaric ensemble) for 100 ps each, with temperature maintained at 300 K (V-rescale thermostat) and pressure at 1 bar (Parrinello–Rahman barostat). Production runs were extended to 100 ns with a 2-fs time step, using the LINCS algorithm to constrain bond lengths. To ensure statistical robustness, three independent replicate simulations were performed for each system. Trajectory analyses (RMSD, RMSF, Rg, SASA) were computed using GROMACS internal tools. Binding free energies were calculated via the MM/PBSA method using the gmx_MMPBSA tool, extracting snapshots from the stable equilibrium phase of the trajectories.

### 4.6. Statistical Analysis

All experiments were performed with at least three independent biological replicates. Data are presented as mean ± standard error (SE). For differential gene expression analysis between the resistant (KC, S627N) and susceptible (a511) lines, raw read counts were normalized and analyzed using the DESeq2 package (v1.38.3) in R (v4.3.2), with genes showing |log_2_ fold change| > 1 and false discovery rate (FDR) < 0.05 considered significantly differentially expressed. KEGG pathway enrichment analysis was performed using the clusterProfiler package (v4.6.2). For comparisons of root activity, binding free energy, and gene expression levels of TaALS family members, Student’s *t*-test or one-way ANOVA followed by Duncan’s multiple range test was applied using R. MD simulation data (RMSD, RMSF, Rg, SASA) were analyzed using GROMACS built-in tools, with statistical significance determined at *p* < 0.05. All figures were generated using the ggplot2 package in R.

## 5. Conclusions

This study demonstrates a complete translational pipeline—from understanding naturally evolved weed resistance to designing predictive crop traits—that integrates forward genetics, multi-omics validation, and high-resolution computational modeling. We developed a novel S627N germplasm and computationally validated the synergistic W548L/S627N double mutant in *TaALS*, revealing an “energy network reprogramming” mechanism that confers strong herbicide resistance while preserving enzymatic function. This work provides both an immediately usable genetic resource and a predictive blueprint for engineering durable, epistasis-informed herbicide tolerance in polyploid wheat. The approach exemplifies a paradigm shift from mimicking natural variation to proactive, simulation-guided crop design.

## Figures and Tables

**Table 1:** Binding free energy components (kcal/mol) calculated by MM/PBSA.

Contribution Components	TaALS-MSM (WT)	TaALS-S627N-MSM	TaALS-W548L-MSM	TaALS-W548L-S627N-MSM
ΔVDWAALS	−54.94 ± 1.21	−52.46 ± 0.85	−56.84 ± 1.15	−54.66 ± 1.04
ΔEEL	−26.87 ± 2.14	−20.25 ± 2.67	−20.12 ± 0.53	−13.57 ± 2.16
ΔEPB	61.00 ± 1.80	56.21 ± 0.76	61.06 ± 1.23	55.09 ± 1.75
ΔENPOLAR	−4.73 ± 0.04	−4.99 ± 0.03	−5.08 ± 0.04	−4.99 ± 0.05
ΔGGAS	−81.81 ± 2.46	−72.71 ± 2.80	−76.97 ± 1.27	−68.23 ± 2.33
ΔGSOLV	56.27 ± 1.80	51.22 ± 0.76	49.98 ± 1.23	50.11 ± 1.75
ΔTOTAL	−25.54 ± 2.05	−21.49 ± 2.91	−20.99 ± 1.77	−18.13 ± 2.76

## Data Availability

The original contributions presented in this study are included in the article/Supplementary Material. Further inquiries can be directed to the corresponding author.

## Supplementary Materials

The following supporting information can be downloaded at: https://www.mdpi.com/article/10.3390/plants15132023/s1. Figure S1. Synergistic reprogramming of the binding energy network in *TaALS-6D*. Per-residue decomposition of binding free energy (kcal/mol) highlights the mechanistic basis of resistance. Figure S2. Transcriptional heatmap of genes in the branched-chain amino acid (BCAA) biosynthesis pathway (KEGG map00290) in wild-type (a511) and S627N mutant (KC) wheat plants. The heatmap displays Z-score-transformed normalized expression levels (FPKM) of genes involved in the valine, leucine, and isoleucine biosynthesis pathway across two genotypes. Each row represents a gene (identified by locus ID), and each column represents an individual biological replicate (n = 3 per genotype). Genes were ordered by hierarchical clustering (Euclidean distance, complete linkage). The color scale indicates relative expression levels, with red denoting higher expression and blue lower expression. The **TaALS-6D** gene (LOC123145358, S627N mutant allele) is marked with an asterisk (*). No systemic up-regulation was observed in the mutant, suggesting the absence of compensatory transcriptional activation in the BCAA biosynthesis pathway. Figure S3. Expression levels of *TaALS* gene family members in wild-type (a511) and S627N mutant (KC) wheat plants. The bar plot shows normalized expression levels (mean ± SD, n = 3 biological replicates) of representative *TaALS* genes, including catalytic subunits (*ALS1*) and small subunits (SSU). The mutant allele **TaALS-6D** (S627N, LOC123145358) is marked with an asterisk (*). No significant difference (Student’s *t*-test, *p* > 0.05) was detected for any ALS gene between genotypes, indicating that the S627N mutation does not trigger feedback regulation of ALS gene expression.

## References

[1] Rodriguez J., Hauvermale A., Carter A., Zuger R., Burke I.C. (2021). An Ala122Thr substitution in the AHAS/ALS gene confers imazamox-resistance in *Aegilops cylindrica*. Pest Manag. Sci..

[2] Zhao N., Yan Y., Wang H., Bai S., Wang Q., Liu W., Wang J. (2018). Acetolactate synthase overexpression in mesosulfuron-methyl-resistant shortawn foxtail (*Alopecurus aequalis* Sobol.): Reference gene selection and herbicide target gene expression analysis. J. Agric. Food Chem..

[3] Tang W., Liu S., Yu X., Yang Y., Zhou X., Lu Y. (2021). The basis of tolerance mechanism to metsulfuron-methyl in *Roegneria kamoji* (Triticeae: Poaceae). Plants.

[4] Zhan Y., Liu H., Cao Z., Chen W., Li Z., Bai L., Pan L. (2022). Comparative analysis of fungal communities between herbicide-resistant and -susceptible *Alopecurus aequalis*. Front. Cell. Infect. Microbiol..

[5] Xu Y., Li S., Hao L., Li X., Zheng M. (2022). Tribenuron-methyl-resistant *Descurainia sophia* L. exhibits negative cross-resistance to imazethapyr conferred by a Pro197Ser mutation in acetolactate synthase and reduced metabolism. Pestic. Biochem. Physiol..

[6] Guo W., Chi Y., Feng L., Tian X., Liu W., Wang J. (2018). Fenoxaprop-P-ethyl and mesosulfuron-methyl resistance status of shortawn foxtail (*Alopecurus aequalis* Sobol.) in eastern China. Pestic. Biochem. Physiol..

[7] Wang N., Bai S., Bei F., Zhao N., Jia S., Jin T., Wang J., Wang H., Liu W. (2022). Resistance to ALS inhibitors conferred by non-target-site resistance mechanisms in *Myosoton aquaticum* L.. Pestic. Biochem. Physiol..

[8] Wang H., Zhang Y., Ren Y., Liu Y., Feng Z., Dong L. (2024). Mechanism of multiple resistance to fenoxaprop-P-ethyl, mesosulfuron-methyl, and isoproturon in *Avena fatua* L. from China. Pestic. Biochem. Physiol..

[9] Yin F., Jiang J., Liao M., Cao H., Huang Z., Zhao N. (2024). Fenoxaprop-P-ethyl, mesosulfuron-ethyl, and isoproturon resistance status in *Beckmannia syzigachne* from wheat fields across Anhui Province, China. Pestic. Biochem. Physiol..

[10] Zhang D., Li X., Bei F., Jin T., Jia S., Bu R., Wang J., Wang H., Liu W. (2022). Investigating the metabolic mesosulfuron-methyl resistance in *Aegilops tauschii* Coss. by transcriptome sequencing combined with the reference genome. J. Agric. Food Chem..

[11] Wang Q., Ge L., Zhao N., Zhang L., You L., Wang D., Liu W., Wang J. (2019). A *Trp-574-Leu* mutation in the acetolactate synthase (ALS) gene of *Lithospermum arvense* L. confers broad-spectrum resistance to ALS inhibitors. Pestic. Biochem. Physiol..

[12] Wang H., Zhang L., Li W., Bai S., Zhang X., Wu C., Liu W., Wang J. (2019). Isolation and expression of acetolactate synthase genes that have a rare mutation in shepherd’s purse (*Capsella bursa-pastoris* (L.) Medik.). Pestic. Biochem. Physiol..

[13] Zhao N., Yan Y., Ge L., Zhu B., Liu W., Wang J. (2019). Target site mutations and cytochrome P450s confer resistance to fenoxaprop-P-ethyl and mesosulfuron-methyl in *Alopecurus aequalis*. Pest Manag. Sci..

[14] Bi Y., Liu W., Guo W., Li L., Yuan G., Du L., Wang J. (2016). Molecular basis of multiple resistance to ACCase- and ALS-inhibiting herbicides in *Alopecurus japonicus* from China. Pestic. Biochem. Physiol..

[15] Yu H., Guo X., Peng L., Li X., Chen J., Cui H. (2023). Target gene mutations endowed cross-resistance to acetolactate synthase-inhibiting herbicides in wild *Brassica juncea*. Pestic. Biochem. Physiol..

[16] Xu X., Zhao B., Li B., Shen B., Qi Z., Wang J., Cui H., Chen S., Wang G., Liu X. (2024). *Trp-574-Leu* mutation and metabolic resistance by cytochrome P450 gene conferred high resistance to ALS-inhibiting herbicides in *Descurainia sophia*. Pestic. Biochem. Physiol..

[17] Adari M.D., Pandian B.A., Gaines T.A., Prasad P.V., Jugulam M. (2024). Confirmation and characterization of the first case of acetolactate synthase (ALS)-inhibitor resistance in Japanese brome (*Bromus japonicus*) in the US. Pest Manag. Sci..

[18] Vijayarajan V.B.A., Torra J., Runge F., de Jong H., van de Belt J., Hennessy M., Forristal P.D. (2025). Confirmation and characterisation of ALS inhibitor resistant *Poa trivialis* from Ireland. Pestic. Biochem. Physiol..

[19] Ribeiro V.H.V., Gallagher J., Mallory-Smith C., Barroso J., Brunharo C.A.C.G. (2025). Multiple origins or widespread gene flow in agricultural fields? Regional population genomics of herbicide resistance in *Bromus tectorum*. Mol. Ecol..

[20] Liu W., Harrison D.K., Chalupska D., Gornicki P., O’Donnell C.C., Adkins S.W., Haselkorn R., Williams R.R. (2007). Single-site mutations in the carboxyltransferase domain of plastid acetyl-CoA carboxylase confer resistance to grass-specific herbicides. Proc. Natl. Acad. Sci. USA.

[21] Li L., Du L., Liu W., Yuan G., Wang J. (2014). Target-site mechanism of ACCase-inhibitors resistance in American sloughgrass (*Beckmannia syzigachne* Steud.) from China. Pestic. Biochem. Physiol..

[22] Wang Z., Jiang M., Yin F., Wang M., Jiang J., Liao M., Cao H., Zhao N. (2024). Metabolism-based nontarget-site mechanism is the main cause of a four-way resistance in shortawn foxtail (*Alopecurus aequalis* Sobol.). J. Agric. Food Chem..

[23] Yanniccari M., Gigón R., Larsen A. (2020). Cytochrome P450 herbicide metabolism as the main mechanism of cross-resistance to ACCase- and ALS-inhibitors in *Lolium* spp. populations from Argentina: A molecular approach in characterization and detection. Front. Plant Sci..

[24] Hada Z., Menchari Y., Rojano-Delgado A.M., Torra J., Menéndez J., Palma-Bautista C., de Prado R., Souissi T. (2021). Point mutations as main resistance mechanism together with P450-based metabolism confer broad resistance to different ALS-inhibiting herbicides in *Glebionis coronaria* from Tunisia. Front. Plant Sci..

[25] Rendina A.R., Felts J.M., Beaudoin J.D., Craig-Kennard A.C., Look L.L., Paraskos S.L., Hagenah J.A. (1988). Kinetic characterization, stereoselectivity, and species selectivity of the inhibition of plant acetyl-CoA carboxylase by the aryloxyphenoxypropionic acid grass herbicides. Arch. Biochem. Biophys..

[26] Zagnitko O., Jelenska J., Tevzadze G., Haselkorn R., Gornicki P. (2001). An isoleucine/leucine residue in the carboxyltransferase domain of acetyl-CoA carboxylase is critical for interaction with aryloxyphenoxypropionate and cyclohexanedione inhibitors. Proc. Natl. Acad. Sci. USA.

[27] Christoffers M.J., Berg M.L., Messersmith C.G. (2002). An isoleucine to leucine mutation in acetyl-CoA carboxylase confers herbicide resistance in wild oat. Genome.

[28] Vázquez-García J.G., de Portugal J., Torra J., Osuna M.D., Palma-Bautista C., Cruz-Hipólito H.E., De Prado R. (2022). Comparison between the mechanisms of Clearfield^®^ wheat and *Lolium rigidum* multiple resistant to acetyl CoA carboxylase and acetolactate synthase inhibitors. Environ. Pollut..

[29] Anthimidou E., Ntoanidou S., Madesis P., Eleftherohorinos I. (2020). Mechanisms of *Lolium rigidum* multiple resistance to ALS- and ACCase-inhibiting herbicides and their impact on plant fitness. Pestic. Biochem. Physiol..

[30] Wang J., Chen J., Li X., Li D., Li Z., Cui H. (2020). *Pro-197-Ser* mutation in ALS and high-level GST activities: Multiple resistance to ALS and ACCase inhibitors in *Beckmannia syzigachne*. Front. Plant Sci..

[31] Zhang R., Liu J., Chai Z., Chen S., Bai Y., Zong Y., Chen K., Li J., Jiang L., Gao C. (2019). Generation of herbicide tolerance traits and a new selectable marker in wheat using base editing. Nat. Plants.

[32] Chen Z., Wang Z., Heng Y., Li J., Pei J., Cao Y., Deng X.W., Ma L. (2021). Generation of a series of mutant lines resistant to imidazolinone by screening an EMS-based mutant library in common wheat. Crop J..

[33] Xu H., Cheng J., Leng Q., Cao R., Su W., Sun L., Xue F., Han Y., Wu R. (2025). Characterization of acetolactate synthase genes and resistance mechanisms of multiple herbicide resistant *Lolium multiflorum*. Plant Physiol. Biochem..

[34] Lei T. (2025). Identification of Wheat Mutants Resistant to Mesosulfuron-Methyl and Study on Their Physiological and Biochemical Characteristics. Master’s Thesis.

[35] Wang W., Yan L., Li J., Zhang C., He Y., Li S., Xia L. (2025). Engineering a robust *Cas12i3* variant-mediated wheat genome editing system. Plant Biotechnol. J..

[36] Ni P., Zhao Y., Zhou X., Liu Z., Huang Z., Ni Z., Sun Q., Zong Y. (2023). Efficient and versatile multiplex prime editing in hexaploid wheat. Genome Biol..

[37] Qi Biodesign (2025). Wheat with mutant TaALS gene for ALS herbicide resistance (*TaALS-4* event). CARBON Newsletter.

[38] Du L. (2016). Fitness Cost Study Based on ACCase Allele Mutations in *Beckmannia syzigachne*. Master’s Thesis.

[39] Papapanagiotou A.P., Paresidou M.I., Kaloumenos N.S., Eleftherohorinos I.G. (2015). ACCase mutations in *Avena sterilis* populations and their impact on plant fitness. Pestic. Biochem. Physiol..

[40] Vila-Aiub M.M., Yu Q., Han H., Powles S.B. (2015). Effect of herbicide resistance endowing *Ile-1781-Leu* and *Asp-2078-Gly* ACCase gene mutations on ACCase kinetics and growth traits in *Lolium rigidum*. J. Exp. Bot..

[41] Frenkel E., Matzrafi M., Rubin B., Peleg Z. (2017). Effects of environmental conditions on the fitness penalty in herbicide resistant *Brachypodium hybridum*. Front. Plant Sci..

[42] Wang F., Xu Y., Li W., Chen Z., Wang J., Fan F., Tao Y., Jiang Y., Zhu Q.-H., Yang J. (2021). Creating a novel herbicide-tolerance OsALS allele using CRISPR/Cas9-mediated gene editing. Crop J..

