# Supplementary material for: From Weed Evolution to Crop Design: A Computational Blueprint for a Novel, Synergistic Herbicide-Resistant Allele in Wheat
Source: Plants (Basel). 2026 Jun 30;15(13):2023. doi: 10.3390/plants15132023 (PMC13364041; doi:10.3390/plants15132023)
Supplement: Supplementary file 1 [file plants-15-02023-s001.zip › plants-4129168-supplementary.pdf]

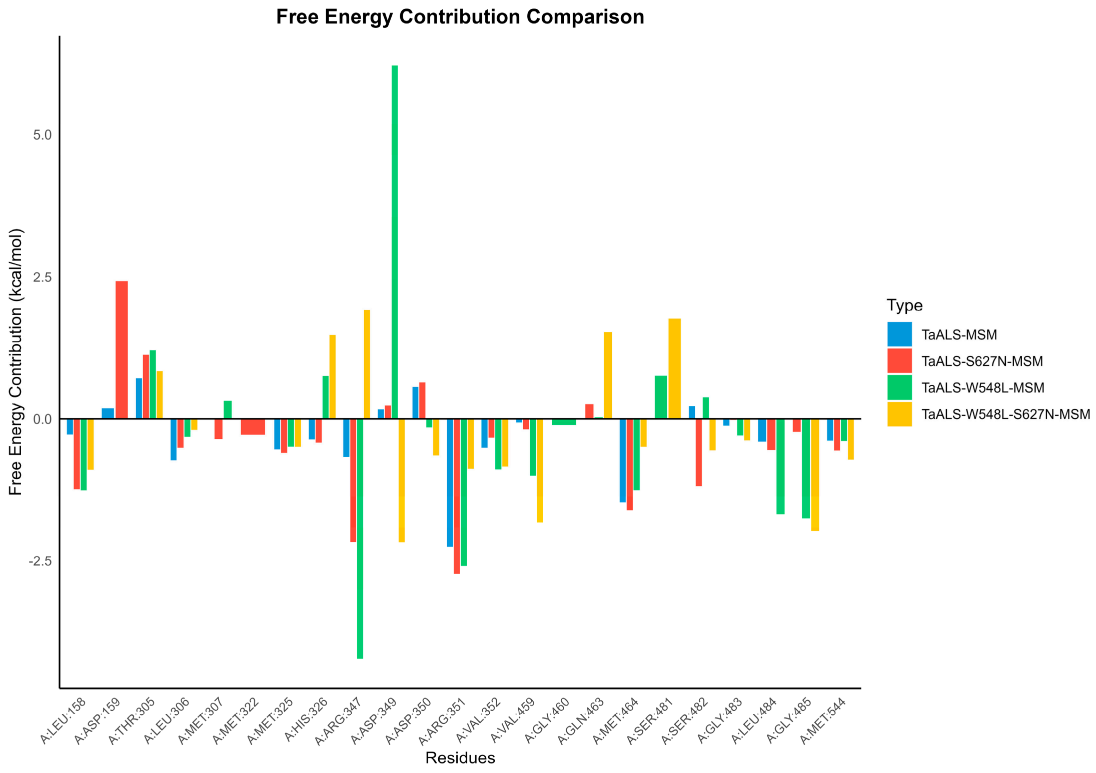

Supplementary Figure S1. Synergistic reprogramming of the binding energy network in *TaALS-6D*. Per-residue decomposition of binding free energy (kcal/mol) highlights the mechanistic basis of resistance.

Wild-type (Blue) and Single Mutants (Red/Green): Generally maintain favorable interactions (negative values) at key residues such as **Arg-347** and **Arg-351**, which constitute the primary herbicide-binding anchor.

Double Mutant (Yellow): Exhibits a unique "epistatic profile" characterized by a dramatic polarity inversion at **Arg-347** (shifting from favorable negative to repulsive positive energy) and the emergence of ***de novo*** energetic penalties at **His-326** and **Gln-463**. These unfavorable energetic peaks confirm that the **W548L/S627N** double mutation induces systemic steric and electrostatic conflicts not present in single mutants, leading to the synergistic destabilization of the herbicide–enzyme complex.

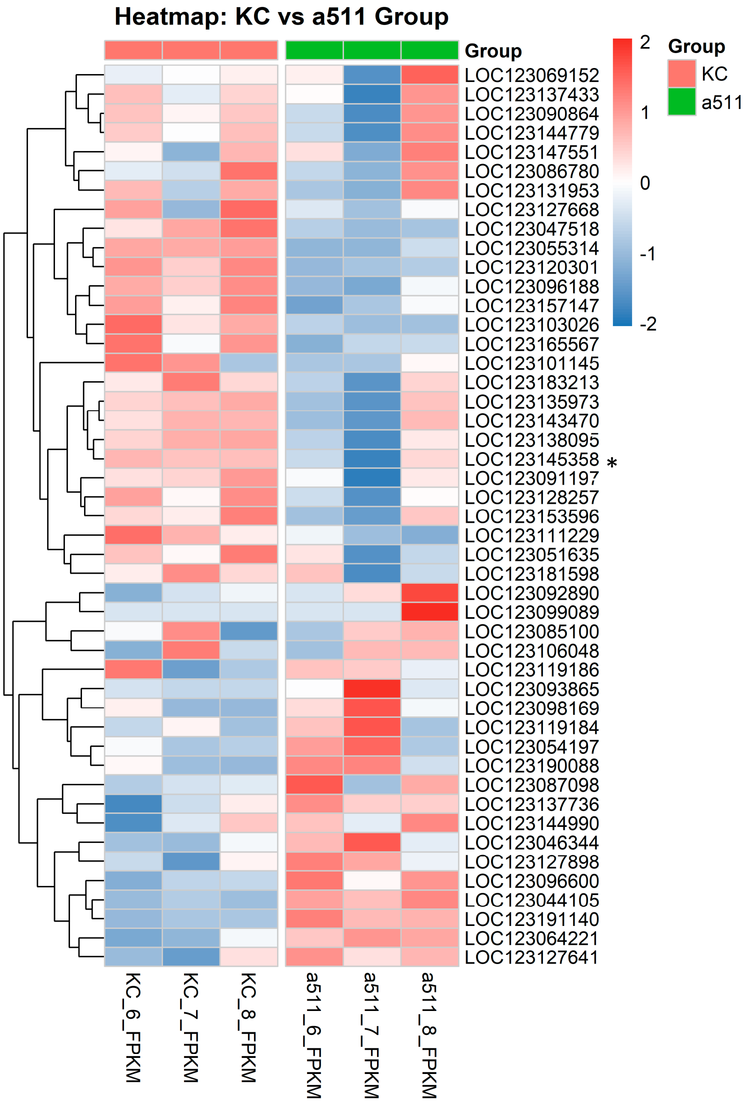

Supplementary Fig. S2. Transcriptional heatmap of genes in the branched-chain amino acid (BCAA) biosynthesis pathway (KEGG map00290) in wild-type (a511) and S627N mutant (KC) wheat plants. The heatmap displays Z-score-transformed normalized expression levels (FPKM) of genes involved in the valine, leucine, and isoleucine biosynthesis pathway across two genotypes. Each row represents a gene (identified by locus ID), and each column represents an individual biological replicate (n = 3 per genotype). Genes were ordered by hierarchical clustering (Euclidean distance, complete linkage). The color scale indicates relative expression levels, with red denoting higher expression and blue lower expression. The **TaALS-6D** gene (LOC123145358, S627N mutant allele) is marked with an asterisk (*). No systemic up-regulation was observed in the mutant, suggesting the absence of compensatory transcriptional activation in the BCAA biosynthesis pathway.

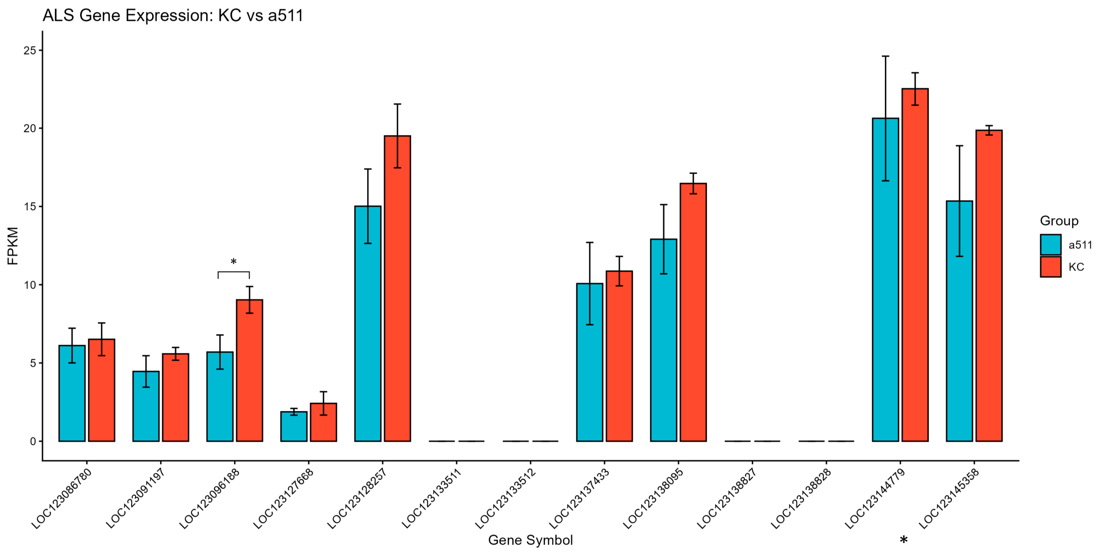

Supplementary Fig. S3. Expression levels of *TaALS* gene family members in wild-type (a511) and S627N mutant (KC) wheat plants. The bar plot shows normalized expression levels (mean ± SD, n = 3 biological replicates) of representative *TaALS* genes, including catalytic subunits (*ALS1*) and small subunits (SSU). The mutant allele **TaALS-6D** (S627N, LOC123145358) is marked with an asterisk (*). No significant difference (Student's t-test, $p > 0.05$) was detected for any ALS gene between genotypes, indicating that the S627N mutation does not trigger feedback regulation of ALS gene expression.
